# Ecological approaches to informing public health policy and risk assessments on emerging vector-borne zoonoses

**DOI:** 10.3134/ehtj.10.001

**Published:** 2010-02-03

**Authors:** JM Medlock, LJ Jameson

**Affiliations:** Medical Entomology and Zoonoses Ecology Group, Microbial Risk Assessment, Health Protection Agency, Salisbury, UK

## Abstract

Pathogens associated with vector-borne zoonoses occur in enzootic cycles within nature. They are driven by a combination of vertebrate host and invertebrate vector population dynamics, which in turn respond to changes in environmental stimuli. Human involvement in these cycles, and hence the occurrence of human disease, is often to act as incidental host. From a public health perspective our ability to better predict human outbreaks of these diseases and prepare intervention and mitigation strategies relies on understanding the natural cycle of pathogen transmission. This requires consideration of, for example, invertebrate and vertebrate ecology and biology, climatology, land use and habitat change. Collectively, these can be referred to as medical entomology and medical ecology. This article reviews the importance for inclusion of such disciplines when assessing the public health risk from vector-borne zoonoses and summarizes the possible future challenges and driving forces for changes in vector status and vector-borne zoonoses emergence, with a particular focus on a UK and European context.

## Introduction

### UK public health risk assessment process

When preparing for and responding to emerging diseases, government officials and scientists are expected to make rapid assessments of risk and inform policy decisions through expert steering groups. Infections such as influenza, SARS, chikungunya and West Nile virus (WNV) have tested our abilities to predict epidemics and provide timely, well-informed advice to policy decision makers.

Since 2003, the Human Animal Infections Risk Surveillance (HAIRS) group, coordinated by the Health Protection Agency (HPA), has provided expert advice on emerging infections to the UK Chief Medical Officer's National Expert Panel on New and Emerging Infections. This group draws in experts largely from the medical and veterinary sectors across the UK^1^ and assesses the risks posed to UK public health by potential emerging infectious diseases threats using a published methodology.^2^ The Medical Entomology and Zoonoses Ecology (MEZE) group at HPA are members of HAIRS, providing input to discussions primarily on vectorborne zoonoses (VBZ). When considering the possible risks of infections that essentially occur within nature (whether it be wildlife, or an invertebrate vector), various considerations are required that may be crucial to the final assessment of public health risk.

### Role of medical ecology and medical entomology in informing risk assessments and policy

A large proportion of emerging human pathogens have a zoonotic source and many of these have wildlife origins or are transmitted via arthropod vectors.^3^–^5^ Given the prospects of climate change, coupled with large-scale ecological and agricultural change, the dynamics of these pathogens will ebb and flow and we are already beginning to see signs of this in Europe.^6^–^14^ With increasing global travel and trade, the geographic distributions of key vectors and their pathogens are equally dynamic.^15^–^19^

Such pathogens occur in enzootic cycles within nature, with humans often acting as incidental hosts. To fully understand and to better predict the dynamics of associated human disease outbreaks, we need to have an intimate knowledge of their ecology and transmission cycle. Without such an understanding, the ability to prepare for, react to, and mitigate the effect of these pathogens is severely hindered. An ecological perspective to the risk assessment process can quickly shed light on what appears initially as an unpredictable complex disease dynamic. In contrast, it can also complicate a seemingly simple disease formula. It may also provide greater opportunities to disrupt the transmission cycle at its weakest point, which in turn can reduce or eliminate the public health burden.

This review does not aim to cover all components of disease emergence such as clinical, virological, bacteriological or immunological changes; although the authors agree such drivers have equal importance. This article will purely review where an ecological/entomological input has been required by MEZE in assessing VBZ risk and, using examples elsewhere in Europe, provide an overview of other ecologically driven disease transmission scenarios that have relevance to informing policy decisions and risk assessment on VBZ; concluding with some discussion of what challenges might arise in the UK over the next 50 years.

No ethical approval for this study was required; an in-depth review of the literature was performed using all relevant VBZ keywords in PubMed. Keywords included: vector-borne, arboviruses, zoonoses, wildlife zoonoses, mosquito, tick, Phlebotomus, Europe, Hantavirus, Dirofilaria, West Nile, Sindbis, Tahyna, Tick-borne encephalitis, CrimeanCongo haemorrhagic fever, chikungunya, *Aedes albopictus, invasive mosquitoes, Ixodes, Dermacentor, Hyalomma, Echinococcus multilocularis, Aedes vexans, Culex pipiens, Rhipicephalus sanguineus*, malaria, disease ecology, mosquitoes and wetlands.

## Vector-borne diseases in twenty-first century Europe

Since the turn of the century, Europe has witnessed unexpected outbreaks of bluetongue virus (BTV) in northern countries,^20^ the continued spread and establishment of the exotic mosquito *A. albopictus* to most of the Mediterranean countries,^21^ and the emergence of chikungunya virus (CHIKV) in Italy.^22^, ^23^ Crimean-Congo haemorrhagic fever virus (CCHFV) and its *Hyalomma* tick vector have also emerged in parts of Turkey, and for the first time caused clinical disease in humans in both Turkey and Greece.^24^, ^25^ New foci of Tick-borne encephalitis virus (TBEV) have been reported further north in Scandinavia.^26^ Usutu virus (USUV) has emerged in Austria,^27^ Hungary^28^ and Spain,^29^ and outbreaks of WNV continue to appear in France,^30^ Hungary,^31^ and Romania,^32^ and emerge in Italy.^33^ One of our commonest vector-borne diseases, Lyme borreliosis, has also seen an increase in incidence rates between 2001 and 2005 in many European countries, with some rates increasing two- or threefold.^34^

The arrival and expansion of BTV, CHIKV and USUV raises questions as to which pathogens and/or vectors will next appear in Europe. Will we see the movement of Rift Valley fever virus (RVFV) from Africa and the Arabian Peninsula? Will CCHFV expand to match the distribution of its vector? Will *Aedes aegypti*, the principal vector of dengue (DENV), spread from Madeira to mainland Portugal and beyond? Looking further ahead perhaps, will we see the re-emergence of malaria and DENV to previously endemic zones in the Mediterranean basin (for example malaria occurred in Italy and large outbreaks of DENV occurred in Greece, although the latter will be dependant on the spread of invasive *Aedes*)? The latter few might seem fanciful, but we should not be complacent. If we learn any lesson from the past, it is time to expect, and prepare, for the unexpected.

## Developing a baseline distribution of invertebrate vectors

A crucial component to any risk assessment on VBZ is knowledge of the distribution and abundance of potential vector species. Without this baseline data it is impossible to assess whether a ‘new finding’ of an unusual or exotic species is significant. It is not generally considered to be cost-effective to have long-term nationwide surveillance programmes for all potential vectors, particularly when they do not currently transmit disease. Occasionally, there are funded programmes that monitor species abundance at a number of key sites across a country and these data are important; however it tells us very little about the nationwide distribution of vector species. It is also of limited use in the development of predictive distribution maps, unless the survey locations are both numerous and reflect the sufficient diversity of ecozones. Recently, in response to the outbreak of CHIKV in Italy, ECDC funded a project to map the European distribution of *A. albopictus*. In less than 3 months, and following concerted European-wide collaboration between entomologists, a map now exists for this exotic mosquito species across more than 20 countries. This has facilitated predictive mapping of their current distribution and the potential consequences of climate change.^35^

Without substantive and ongoing funded programmes to map vector species, the sustainable and affordable option is to capitalize on and engage the volunteer recording network. In the UK, naturalists and scientists have been recording species occurrence since the eighteenth century, with 15 million records of 12,000 species currently held by the Biological Records Centre.^36^ Of course much of the data relate to non-vector species of biodiversity interest, but it illustrates the potential of thousands of invertebrate recorders willing to submit data. In 2005, MEZE established a tick recording scheme,^37^ and has now received 1000 records and >4000 ticks from across the UK.^38^ Added to 4500 historical records, this provides our best data set for ticks, and is now being used to facilitate geo-spatial mapping.A similar initiative for mosquitoes has also been running since 2005.^39^ Although this kind of data are often regarded as ‘*ad hoc*’ and unsystematic, they inform public and veterinary health authorities where nuisance-biting vectors occur, provide an early warning system for exotic or expanding species and provide a bank of samples for pathogen analysis. Furthermore, it is very cost-effective and requires the time of the scheme coordinators to perform and validate identifications.

## Understanding vector behaviour and classification

For non-medical entomologists, a list of potential vector species and their distribution can be misleading without specific knowledge on their biology, ecology, classification and vector competence. Recent examples of this relate to potential mosquito vectors of WNV and RVFV. During the outbreak of WNV in North America, C. *pipiens* was quickly implicated as a key vector in the eastern states.^40^ In the UK it exists as two separate forms, an exclusive ornithophagic *pipiens* form, and predominantly mammalophilic (also partly ornithophilic) *molestus* form.^41^ These two forms adopt hugely different behaviour and aquatic habitats and do not, as far as we are aware, hybridize. In the USA, however they do and some studies have found that 40% of C. *pipiens* are hybrids and consequently feed on both birds and humans, thus driving the transmission cycle.^42^ In the UK then, although our *pipiens* form might be an important enzootic vector, it is unlikely to be a human vector despite its synanthropic container breeding habits and its propensity for over-wintering in the cool recesses of houses.^43^ The status of the British forms of this mosquito is still a topic of much debate in DNA barcoding circles.

Similarly, one of the primary vectors of RVFV is *A. vexans*, which also exists as a species complex including subspecies *vexans* and *arabiensis*; for example, *A. vexans arabiensis* is an important vector in Senegal and Saudi Arabia. *A. vexans* occurs also in parts of western Europe^44^, ^45^ and in a recent study involving MEZE in Epping Forest was found to cause nuisance biting close to the London Olympics village site.^46^ Should this be a concern, and should we assess its taxonomy and vector competence?

A number of studies, including those by MEZE on WNV and SINV, have taken an entomological perspective to risk assessment, by reviewing the status, behaviour and distribution of putative vector species.^43^, ^47^–^49^ This rapidly identifies which species could function as vectors, their likely aquatic breeding sites (and hence synanthropic/sylvatic issues) and, if known, their biting behaviour. Often important data are lacking, particularly information on the host preferences (for example bird or horse biting) of certain vector species, which can be significant in understanding transmission cycles and disease risk. Such reviews also help to target research gaps for field entomology and ecology and to identify key species for vector competence studies and viral isolations.

## Vertebrate host dynamics and vector-borne infections

Knowledge of the involvement of vertebrate species as reservoirs or amplifying species can rapidly affect the level of risk attributed to an emerging disease. Even with an abundant vector population, the absence of key vertebrate hosts for all or part of the year is significant. Studies from Sweden and Finland on Sindbis virus (SINV; the cause of Ockelbo and Pogosta diseases in humans) illustrate this point very well.^50^, ^51^ SINV exists within a bird–mosquito cycle. In Sweden, it cycles between passerines and *Culex* mosquitoes, with *Aedes* providing the link to humans,^52^–^54^ and in Finland vertebrate hosts are tetraonid birds.^55^ Following reports of antibodies to this virus in the UK^56^ where all key putative mosquito vectors occur,^43^ concerns were raised over possible endemic transmission, and an ecological assessment was performed by MEZE.^48^

This work concluded that particular tetraonid bird species *(Tetrao urogallus, Tetrao tetrao)* that amplify the virus in Finland^54^ also occur in Britain, but only in a few Scottish forest and moorland sites. Both species are rare and consequently unlikely to be important in amplifying the virus or contributing significantly to virus cycles in birds. Given the ubiquity of passerines in the UK however, and their general role as reservoirs in Sweden, it is suggested that perhaps a lack of reservoir hosts might not be a limiting factor in transmission. However, Swedish studies found that human outbreaks of Ockelbo disease appeared to be linked to the occurrence of certain migratory thrush species.^55^, ^57^ Redwing *(Turdus iliacus*), fieldfare *(T. pilaris)* and song thrush *(T. philomelos)* were found to have high prevalence rates for virus and were implicated as key amplifying hosts. These species breed in Sweden in the summer and have large numbers of fledgelings during the main peak in mosquito activity. In autumn, redwing and fieldfare fly south to spend the winter in Britain, usually after the peak in mosquitoes, and hence are unlikely to contribute significantly to virus amplification. An understanding of the role of different hosts and bird life histories can significantly change our perception of disease risk.

## Impact of weather and climate on vector, host and pathogen dynamics

### Temperature-dependent distribution and seasonal activity of vectors

Many aspects of ecological systems are affected by climatic changes, particularly the effect of temperature. The expected change in summer and winter temperature as a consequence of climate change will change vector behaviour and seasonality, and often this requires a longer-term assessment of risk. For example, phlebotomine sandflies are acutely responsive to temperature changes and new reports of their survival further north (that is, north of the Mediterranean) have been reported in Germany.^58^ *Phlebotomus* sandflies are vectors of canine and human leishmaniasis, and there is a genuine concern that travelling dogs that are returning to the UK with *Leishmania* parasites could create a reservoir of infection, which in the event of sandflies establishing could lead to local cycles.^59^ In the absence of current surveillance for sandflies, MEZE are working with expert dipterists to develop an early warning system.

Mosquitoes use temperature as a seasonal cue, and along with photoperiodic cues, these have been used to model their seasonal activity. For temperate strains of exotic species, like *A. albopictus*, models, developed by MEZE and others, have assessed areas for establishment and seasonal activity in the event of an importation,^60^, ^61^ thus providing an insight into possible areas for nuisance biting and to target surveillance.

Changes in climate also affect the seasonality and/or distribution of ticks, with evidence of a gradual northerly spread of *I*. *ricinus* in response to consecutive mild winters in Scandinavia.^62^ For *R.* *sanguineus*, it has even been suggested that host preference is altered in response to temperature, with the affinity of immature *R.* *sanguineus* for humans apparently increased during short periods of warmer weather (>25°C) in France.^63^ Given the recent discussion over permitting dogs with tick treatment to travel to the UK, the influence of climate is significant when advising on the potential for establishment of R. *sanguineus* in the UK.^64^

### Effects of temperature on extrinsic incubation of pathogens

Many arthropod-borne pathogens undergo a period of development through the vector, known as the extrinsic incubation period. This period, particularly for parasites like *Plasmodium* and *Dirofilaria*, is temperature dependent, with parasite development enhanced above a set temperature threshold. Laboratory and field studies on these two parasites in the USA on *Dirofilaria* 65–68 and in Russia on *Plasmodium* 69 have aided MEZE and others in our predictions of the likely period of incubation of their larval stages through a hypothetical mosquito.^70^–^72^ This enables us to predict currently (that is based on current weather patterns) how many days the development of the parasite through to the infective third larval stage might be permissible. This information is making us re-assess our opinions on whether northern Europe is too cold to prevent or limit transmission of seemingly tropical or Mediterranean pathogens. However, other factors, such as availability of infected hosts, abundance of receptive vectors and the impact of treatments, need to be considered.

### Effects of sequential climate events on reservoir host dynamics

Studies on the impact of weather patterns on non-arthropod wildlife zoonotic infections, for example, those caused by Puumala virus, have shown that interpreting sequential climate events can allow prediction to be made about changes in the abundance of reservoir hosts (that is, *Myodes glareolus* voles)^73^ and outbreaks of human disease (for example *Nephropathia epidemica)*.^74^, ^75^ The essence of this approach taken by MEZE is to predict the peak years in tree nut production that drive the peaks of vole cycles. In the case of beech *(Fagus sylvatica)*, a sequence of climate events over 2–3 years stimulates a synchronous increase in seed/nut production; known as a ‘masting event’. The vole population consequently peaks the following year and stimulates an increased circulation of virus, which leads to outbreaks of human disease. Equally this has implications for other pathogens like *B.* *burgdorferi* that circulates in rodent populations.

### Societal change masking climate change

These relatively simple uses of weather and climate data are providing important insights into the effects of climate change and possible changes in disease risk. However, climate should not be taken in isolation. Recent studies on TBEV in eastern Europe have shown that climate changes may be masking underlying societal changes, suggesting that economicand climate-influenced factors are combining to impact on the transmission of TBEV. For example, in the Baltic states there has been an increase in outdoor activities as a consequence of increased wealth since the decline of Communism; a decline in industry has changed the amount of local solar radiation, adapting the local climate, thus favouring co-incident seasonality of tick stages, thus increasing co-feeding transmission, and hence increased exposure to virus.^76^–^79^ Indeed, the concept of co-feeding of immature *I. ricinus* in determining TBEV endemic foci in central Europe was one of the first studies to show the complexities of tick seasonality and behaviour of disease transmission cycles.^80^, ^81^

## Human impacts: trade and travel

The impact of global trade and human travel on vector-borne disease is well established. The best examples are the worldwide spread of exotic mosquitoes on used tyres (for example *A. albopictus, Ochlerotatus japonicus, A. aegypti*), and the importation of CHIKV by a human traveller from India to Italy.^82^ Movement of animals and potentially infected or infested animals often goes unnoticed and can have significant implications. There is still much debate about causes of the emergence of WNV in North America with introduction of the virus through imported infected birds being one theory.^83^, ^84^ The involvement of livestock movement in pathogen spread was suggested as one of the factors involved in the emergence of RVFV in Saudi Arabia; imported from Africa.^85^

A recent example studied by MEZE has identified a different implication to the movement of wildlife zoonoses through pet travel. There is currently much debate in the UK over the relaxation of tapeworm control in dogs returning to the UK from continental Europe.^86^–^88^ Current controls are in place to maintain the UK free of *E. multilocularis*,^89^ which is an emerging urban zoonosis in continental Europe^90^ exacerbated by the urban fox population.^91^ This parasite occurs within enzootic cycles between voles (including the water vole, *Arvicola terrestris*) and the fox (*Vulpes vulpes*). A recent assessment by MEZE^92^ has highlighted that in addition to the public health issues, the importation of this parasite by dogs into the UK could undermine current UK wildlife policy^93^ and legislation^94^ that promote the protection of water voles and enhance both their habitats and the grassland habitats of another potential reservoir host, the field vole *(Microtus agrestis).* Coupled with an established urban fox population there is the prospect that an inadvertent importation of this parasite on health risk assessment grounds could conflict with environmental impact assessment, thus undermining green initiatives and establishing enzootic cycles that would be difficult to eradicate. MEZE findings^92^ also question whether such an act could be at odds with EU legislation^95^, ^96^ on non-native species and their effect on native fauna.

## Multiple drivers for emergence

Although not discussed in depth here, it is now recognized that viral mutation and a change in vector specificity were important drivers in the large-scale epidemics of CHIKV in the Indian Ocean region.^97^ The subsequent outbreak of CHIKV in Italy further showed how a combination of factors such as global trade in tyres and global travel of infected individuals can act in synergy to drive the emergence of an exotic virus vectored by an exotic mosquito.

Current unpublished MEZE work in Kosova is showing that multiple drivers may be driving cycles of CCHFV and *Hyalomma* ticks. It appears that a combination of the decline in agriculture (hence the encroachment of scrub and grassland close to the village fringe), the dramatic increase in reservoir host numbers as a possible consequence of predator decline, coupled with the importation of virus-naive livestock (following the recent conflict), could all be contributory factors to increasing the abundance and public exposure of *Hyalomma* and enhancing the circulation of CCHFV in ticks in parts of rural Kosova.

## Possible driving forces for future change in VBZ in the UK

A key component of ‘horizon scanning’ is looking for future issues. Arthropod disease vectors such as mosquitoes and ticks are, like all invertebrates, acutely responsive to changes in their environment, with an ability to evolve to local climate rapidly. There has been much discussion over the role of weather and climate on infectious diseases, with some models able to predict future epidemics and new foci.Notable examples for RVFV,^98^ meningitis,^99^ malaria^100^ and TBEV^101^ have included environmental parameters such as rainfall, wind direction, temperature and seasonal climate. Much less discussion has focussed on non-climate-related environmental changes, or environmental changes as a consequence of adaptation to climate change. Some examples from a UK context are briefly discussed here.

Biodiversity-enriching strategies are likely to have some impact on vector survival, activity and spread. Recent UK biodiversity initiatives promote habitat connectivity by encouraging wildlife corridors on a national^102^, ^103^ and a local^104^ scale. These strategies aim to de-fragment habitats, allowing more vulnerable species of nature conservation concern to move between habitats and hence respond to climatic changes. Often this landscape ecology approach encourages the parcelling of the landscape into habitat patches that form mosaics of biotypes linked by an ecological matrix connecting the fragments of species-rich habitats.

It is not known what effect this will have on disease vectors. Undoubtedly it provides a more robust network for movement of vector hosts (for example, deer, small mammals) and an increase of ecotonal habitat (that is, habitat at the interface of two habitats, for example grassland and woodland) so often favoured by *I. ricinus* ticks and their hosts (for example pheasant). Some authors argue that habitat de-fragmentation can increase the transmission of zoonotic and anthropozoonotic diseases by bringing humans, livestock, wildlife and their pathogens ever closer together.^105^–^107^ MEZE studies have shown that the spatial heterogeneity of ticks within a varied landscape is predictable,^108^ and more recent MEZE work,^109^ looking at the impact of woodland ride management on *I. ricinus*, has found that hot spots of questing tick activity are predictable within lowland woodlands, and that ride and bracken management can be planned to mitigate the public's exposure to ticks and limit the tick's survival in the mat layer.

In the UK, part of enhancing habitat connectivity has involved paying environmental subsides to encourage farmers to create field margins and re-instate hedgerows, ponds and ditches.^110^ Furthermore, on the fragments of relict calcareous grassland, which remain among the farmland, traditional grazing strategies are being re-established to ensure that a diverse sward creates a diverse flora supporting specialist invertebrates. There is little known about the impact of these strategies on tick survival and movement. Ongoing MEZE work suggests that field margins can act as *I. ricinus* habitats, and there are suggestions (anecdotal and published) that the movement of livestock can lead to the establishment of ticks in new geographical foci.^111^

Allied to terrestrial UK initiatives are nationwide plans to return large parts of the UK's drained agricultural land to wetland, fen and salt marsh (for example UK Wetland Vision).^112^ This is likely to have huge biodiversity benefits by making the wildlife of existing wetlands more sustainable, increasing the general available resource for wildlife as well as providing better management of flood waters in low lying areas, river valleys and flood alleviation schemes in urban areas. It would seem prudent therefore that habitat creation plans include a health risk assessment component to the management plan, focusing on the possible effects on mosquito habitats and nuisance biting/disease transmission. MEZE are currently working on such an approach with the Great Fen Project; an environmental project that plans to restore >3000 hectares of arable farmland in Cambridge-shire back to fen, wetland and wet woodland. Some question whether new wetland means more mosquitoes,^113^ others argue that focus should be given to managing habitats for the vectors’ natural predators as they may have some role in keeping vector populations at manageable levels.^114^ Wetland initiatives along the coast have other motives, such as creating salt marshes to help defend an eroding coastline (for example, in eastern England) or by inadvertently adapting coastal habitats to harness the power of tides, such as the Severn Tidal Barrage.^115^ Undoubtedly, the creation of new salt marshes could provide new habitats for our brackish water *Anopheles sp*., which lost their habitats, and the malaria they transmitted, following wide-scale drainage in the nineteenth century.^116^–^119^ However, the arguments here for the return of malaria are more complex and have been discussed elsewhere.^120^

In urban areas, changes are also occurring, whether it is an extension of urban areas into the peri-urban fringe, the re-wilding and greening of urban habitats, restoration of industrialized river corridors or wildlife gardening. MEZE have received several reports to its tick recording scheme regarding high levels of tick activity in residential gardens, mostly attributable to the encouragement of wildlife to feed and shelter close to human habitation.38 Often we cannot pick and choose the wildlife we attract. Although we may wish to see feeding birds and browsing deer, we also have to accept that we may also attract rats and ticks.

Furthermore, given the recent spread of the container habitat mosquito *A. albopictus* in Europe, and its involvement in CHIKV cycles,^35^, ^121^ there is good reason to be cautious about the management of water in domestic settings. Currently water butts provide a suitable habitat for mosquito species such as C. *pipiens* and *Culiseta annulata*, which are not currently involved as disease vectors in the UK. Following the introduction of an exotic vector species that can exploit these habitats, a re-assessment of our domestic husbandry regarding conserving water will be required, as occurs in Italy.^122^

In conclusion, possible environmental change may have significant impacts on the future changes in VBZ. It is paramount therefore that medical entomologists and epidemiologists work with ecologists, environmental scientists and land managers to pre-empt, prepare for and mitigate the possible changes in VBZ through environmental management. Achieving this preparedness and establishing routes of communication take time, but it should not be left to hastily prepared responses to nuisance biting or outbreaks of disease. A balanced evidence-based opinion is required whereby public health matters are considered, but not in an alarmist way that undermines biodiversity and water management objectives. We have the opportunity as public health practitioners to work with the environment sector to ensure that an increase in biodiversity does not lead to emergence or re-emergence of VBZ.
